# Multispectral LIF-Based Standoff Detection System for the Classification of CBE Hazards by Spectral and Temporal Features

**DOI:** 10.3390/s20092524

**Published:** 2020-04-29

**Authors:** Lea Fellner, Marian Kraus, Florian Gebert, Arne Walter, Frank Duschek

**Affiliations:** German Aerospace Center (DLR), Institute of Technical Physics, Im Langen Grund 1, 74239 Hardthausen, Germany; lea.fellner@dlr.de (L.F.); marian.kraus@dlr.de (M.K.); florian@gebert-bs.de (F.G.); Arne.Walter@dlr.de (A.W.)

**Keywords:** laser-induced fluorescence (LIF), fluorescence lifetimes, standoff detection, biological agents, early warning

## Abstract

Laser-induced fluorescence (LIF) is a well-established technique for monitoring chemical processes and for the standoff detection of biological substances because of its simple technical implementation and high sensitivity. Frequently, standoff LIF spectra from large molecules and bio-agents are only slightly structured and a gain of deeper information, such as classification, let alone identification, might become challenging. Improving the LIF technology by recording spectral and additionally time-resolved fluorescence emission, a significant gain of information can be achieved. This work presents results from a LIF based detection system and an analysis of the influence of time-resolved data on the classification accuracy. A multi-wavelength sub-nanosecond laser source is used to acquire spectral and time-resolved data from a standoff distance of 3.5 m. The data set contains data from seven different bacterial species and six types of oil. Classification is performed with a decision tree algorithm separately for spectral data, time-resolved data and the combination of both. The first findings show a valuable contribution of time-resolved fluorescence data to the classification of the investigated chemical and biological agents to their species level. Temporal and spectral data have been proven as partly complementary. The classification accuracy is increased from 86% for spectral data only to more than 92%.

## 1. Introduction

A release of chemical or biological hazards requires a fast detection to minimize the exposure to first responders and to initiate appropriate countermeasures, i.e., suitable, purposeful and fast. Standoff detection techniques are highly predestined for operations in risky and harsh environments: these methods provide information in almost real-time from safe distances and offer additional support to responders and chemical or biochemical laboratory staff. Among the manifold available standoff detection systems, most prominent are passive sensors (especially in the infrared) [1,2] and systems with active illumination of the target substance [3]. For active standoff applications, lasers are often the first choice as illuminator and initiator for physical and/or chemical substance-specific processes in the sample. Laser-induced breakdown spectroscopy [4] reveals the elementary and structural composition of CBE (chemical, biological and explosive) agents and has found many applications for the detection of chemicals and explosives [5,6] and bacteria e.g., in Ref. [7]. In terms of standoff application (like environmental monitoring or operation by first responders), LIBS has limited applications due to laser-safety issues and due to increased requirements to the optical setup at large distances. The highly substance-specific vibrational spectroscopic methods, Raman [8,9] and mid-infrared absorption spectroscopy [10,11,12,13,14,15] are well-suited for chemicals and explosives as well as—for in-situ Raman spectroscopy and microscopy—for identification of biological matter and single bacterial cells [16,17]. Though standoff distances have been increased continuously for chemical and explosive detection applying mid-infrared laser excitation and photo-acoustic or photo-thermal effects, main applications for bio-detection at large standoff distances seem to be challenging in this wavelength region. Laser-induced fluorescence (LIF) [18,19,20,21,22,23] is commonly used as a standard technique in biological, quantitative and qualitative analyses. In comparison to the techniques above, LIF’s main advantage is the combination of high sensitivity and low pulse energies of the exciting laser at low to moderate costs. Different LIF-based early warning systems (in the standoff and air sampling configuration) have been reviewed by Li et al. and Huffman et al. in recent papers [24,25]. In many developments [26,27], LIF is measured after excitation by radiation of a single or dual wavelength at standoff distances even beyond hundreds of meters, combined with elastic scattering LIDAR (light detection and ranging) for cloud monitoring. Obtained spectral LIF data (and extracted information) still seem—compared to above non-LIF methods—unstructured and reveal limited information as mentioned by Johnson et al. [28].

Thus, an important question to be answered in this work is how to enhance the specificity of the LIF technology for standoff applications. The suggested approach is the following: Fluorescence emission can be regarded as a time-dependent process. The duration of this fluorescence process can be described by fluorescence lifetimes (which are characteristic properties of a molecule and its environment) and traditionally by fluorescence spectra. A basic question in this work is to evaluate the correlation between time-resolved and spectral information in fluorescence signals, i.e., the extension of its orthogonality.

Following a development by Gebert et al. [29], this work aims at including simultaneous measurements of fluorescence lifetimes into acquired spectral data sets. Recent results acquired with an improved setup [29] are presented:

- Standoff LIF measurement data of six selected chemicals and seven types of bacteria
- Analysis and classification of LIF spectral data, only
- Analysis and classification of fluorescence lifetime data (emission at 460 nm), only
- Combined analysis and classification of LIF spectral data and fluorescence lifetime data

The evaluation of the data focuses on additional information gain from these time-dependent fluorescence measurements of biological agents and an improvement of the classification of the substance level.

## 2. Materials and Methods

### 2.1. LIF Detection System

**Figure 1:**
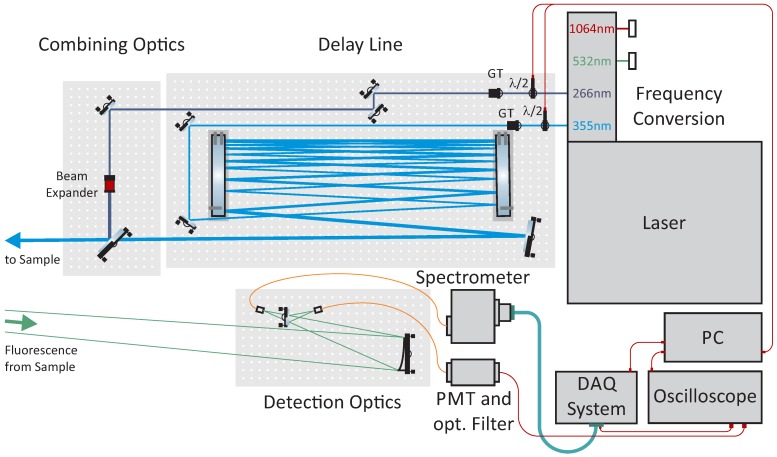
Scheme for standoff laser-induced fluorescence (LIF) detection setup including dual-wavelength excitation and the acquisition of spectral and time-resolved fluorescence (GT: Glan–Taylor prism, PMT: photomuliplier tube).

The presented standoff detection system (see Figure 1) utilizes LIF signals retrieved after excitation with laser pulses of two different UV excitation wavelengths, 266 nm and 355 nm. An Innolas Picolo Magna EVO III laser source provided pulses of both wavelengths with a pulse width below 700 ps at 100 Hz. An electronically-controllable waveplate (λ/2) polarizer (GT) setup is used to adjust the laser pulse energy to the fluorescence strength of each sample. In order to irradiate the samples successively, an optical delay line with a pathlength of ~25 m was used for separating the two laser pulses by up to ~80 ns. The delay line consists of two tilted mirrors at a distance of 80 cm with multipass reflections for the 355 nm laser pulses. The geometrically co-linear laser pulses were pointed onto the sample, with a delay of each 266 nm pulse of ~80 ns to the 355 nm pulse. This allows for a fully separated data acquisition of the returned fluorescence signals. For the first operation and investigations in a laboratory environment with limited space, the collection optics have been designed for a short-range standoff detection distance. Fluorescence return signals were collected by an off-axis parabolic mirror (10 cm diameter) and guided into the spectrometer by a fiber optical cable. For fast data acquisition, a spectrometer (Hamamatsu A10766) with 10 cm focal length and 600 grooves/mm UV grating with a blaze wavelength of 400 nm and a photomultiplier tube (PMT) array (Hamamatsu H7260-04) with 32 channels (used for each excitation wavelength) and a spectral resolution of ~14 nm were used. An electronic integrator (Vertilon IQSP482) accumulates the charge produced in the PMT array over 50 ns. In order to enhance the quality of time-resolved fluorescence signals, an additional PMT with a band-pass filter transmitting from 455–465 nm was used to acquire time-resolved data from both excitation wavelengths simultaneously. Compared to a previously presented system based on 280 and 355 nm excitation (Ref. [30]), the instrument in this work combined temporal and spectral LIF data. The system utilized a fast data acquisition with higher repetition rates focusing on essential fluorescence features. The compact spectrometer with a sensitive PMT detector array allows for low power laser operation in the eye-safe regime.

All results in this work have been gained from 5 × 100 measurements for each sample with pulse energies ranging from 2 nJ to about 100 μJ and at a standoff distance of 3.5 m. Pulse energies were adjusted for a comparable fluorescence yield. Within the utilized range, no non-linear effects and no changes in time-resolved data were observed.

### 2.2. Biological and Chemical Samples

The investigated bacterial strains *Bacillus atrophaeus* (DSM 7264), *Bacillus thuringiensis* (DSM 6102), *Burkholderia fungorum* (DSM 17061), *Burkholderia pyrrocinia* (DSM 10685), *Micrococcus luteus* (DSM 20030), *Oligella urethralis* (DSM 7531) and *Yersinia aldovae* (DSM 18303) were obtained from the Leibniz Institute DSMZ—German Collection of Microorganisms and Cell Culture. Cultivation of the bacterial species was carried out on nutrient agar 1 (Sifin, Berlin, Germany) at 37 °C for 24 h. Colony material was harvested and suspended in phosphate-buffered saline (PBS; Dulbecco’s Phosphate-Buffered Saline modified, without calcium, Sigma-Aldrich, Darmstadt, Germany). The concentrations of the bacterial suspensions were adjusted to an optical density of 2–25 McFarland by using a DEN-1 Densitometer (Grant Instruments, Cambridge, UK). Measurements were conducted in 3500 μL cuvettes (117-QS, Hellma GmbH and Co. KG, Müllheim, Germany), while the samples were continuously stirred with a magnetic bar and stirrer (IKA color squid, IKA-Werke GmbH and Co. KG, Staufen, Germany). Diesel samples were taken from two local gas stations (Deutsche Tamoil GmbH (HEM) and Kaufland GmbH). The tested motor oils are available as Shell Helix 5W-40 (Shell Deutschland Oil GmbH, Hamburg, Germany) and Liqui Moly Marine 15W-40 (Liqui Moly GmbH, Ulm, Germany). Kerosene was obtained from Merck KGaA (Darmstadt, Germany) and Anderol 555 from the manufacturer Anderol (Venlo, Netherlands). All oils were measured undiluted as pure substances.

### 2.3. Data Processing and Evaluation

In addition to the LIF data acquisition procedure including spectral and time-resolved data, the described system contains the data preprocessing and classification in order to get an instantaneous assignment of the measured sample after a few seconds. As already described in Ref. [31] signals below the excitation wavelengths are discarded as well as the working range of the notch filter which blocks the second excitation wavelength (355 nm) in the first signal (excited by 266 nm). Raman peaks may occur in adjacent regions to the excitation wavelength. Raman signals from the solvent water at 3200–3600 cm${}^{-1}$ (peak positions at 292 and 405 nm for excitation at 266 and 355 nm, respectively) are eliminated from the data. Furthermore, some spectra of low intensity show a signal around 532 nm, caused by a remaining signal of the second harmonic output of the Nd:YAG laser. So for both excitation wavelengths the according two channels are set to zero. For reasons of comparability, the spectra are scaled by setting the maxima to 1 and the minima to 0.

Each time-resolved fluorescence signal is despiked, smoothed and also scaled from 0 to 1. Due to a minor temporal jitter, the exact peak position is not fixed and has to be determined. Here, peaks are defined with a minimum scaled intensity of 0.9 and a minimal full width at half maximum (FWHM) of 1 ns. From the found peak positions, the consecutive six-time intervals of length 1, 1, 1, 2, 3 and 5 ns are binned to their medians, creating six temporal features. The subsequent data analysis and classification procedure itself has been published in Ref. [23]. The C5.0 decision tree algorithm is applied in this work [32]. This procedure for evaluating time-resolved data is fast and robust, since it avoids fitting and de-convolution steps for the retrieval of fluorescence lifetimes in complex biological systems.

## 3. Results and Discussion

**Figure 2:**
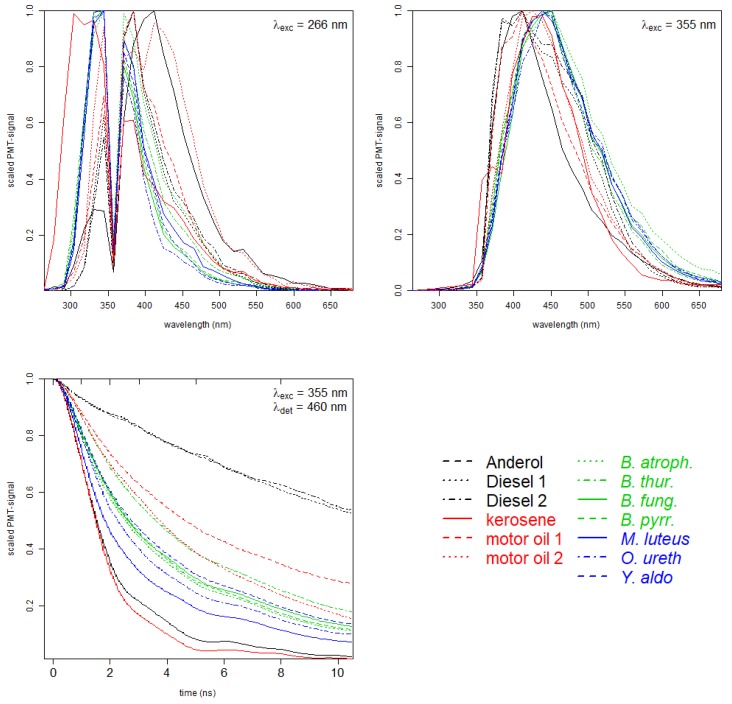
Measured dataset for seven different bacteria and six commercially available oils. **Upper** graphs: Fluorescence spectra with $\lambda_{exc}=266$ nm and $\lambda_{exc}=355$ nm, respectively. **Lower** graph: time-resolved data ($\lambda_{exc}=355$ nm, $\lambda_{det}=460$ nm).

Measured spectral and time-resolved data are presented in the graphical line-up in Figure 2 for all examined bacteria and oil types. For both excitation wavelengths, 266 nm in the left panel and 355 nm in the right panel of Figure 2, the green and blue curves of bacteria can clearly be distinguished from the black and red curves of oil samples. Within the group of bacteria, the spectral curves reveal slight differences for some samples (especially for *Burkholderia fungorum*, *Burkholderia pyrrocinia* and *Yersinia aldovae*), as well for the two types of Diesel.

Fluorescence spectra in the upper panels clearly indicate differences between the fluorescence signatures. The graph on the lower left represents the measured time-dependent PMT signals with an excitation at $\lambda_{exc}=355$ nm and a detected emission in the spectral range from 455 and 465 nm, for simplicity $\lambda_{det}=460$ nm. This detection window was chosen because it provides a high fluorescence intensity for all samples for excitation at $\lambda_{exc}=355$ nm. In this work, time-resolved signals for excitation at $\lambda_{exc}=266$ nm were neglected due to a low signal to noise ratio. Generally, especially for samples like bacteria which contain several chromophores, time-resolved fluorescence from different excitation wavelengths may provide additional information and will be considered for future applications. The curves show a distinct characteristic decay time for all the samples, with long lifetimes for the diesel samples ranging down to short lifetimes for kerosene and Anderol. Within the bacteria samples, the spread is comparably low, especially to *Burkholderia* and *Yersinia aldovae* which can hardly be distinguished.

In order to check if spectral and temporal data provide orthogonal data or are redundant, a decision tree-based classification has been performed on the recorded spectral data, temporal data and the combined data set. The developed classification model is based on 500 spectral and fluorescence decay signals for each sample and excitation wavelength. Randomized samples for training (75%) and testing (25%) were used, following standard cross-validation procedures.

Table 1 summarizes the classification results for the test data.

The upper part Ⓐ contains the confusion matrix for the classified spectral data with an accuracy A=TP/(TP+FP+TN+FN)=86.5% (TP are the true positives, i.e., correctly assigned samples, FP are the false positives, i.e., incorrectly assigned samples, TN are the true negatives and FN are the false negatives). In the middle part Ⓑ, the predictions for the test set are listed if only time-resolved information is used. With full information Ⓒ, spectral and time-resolved, the classification results in an accuracy A=92.6% and for each substance, at least 96 out of 125 measurements are correctly assigned in the test.

From the confusion matrices Ⓐ, Ⓑ, Ⓒ in Table 1 generated after classification of the measured fluorescence data, full information on predictions can be extracted.

The matrices are discussed for the most unsuccessful predictions based on spectral data Ⓐ of the Burholderia, Oligella and Yersinia species (prediction error > 10%): For spectral data Ⓐ, *Yersinia aldovae* was correctly predicted with 64.0% and falsely as *Burkholderia pyrrocini* with 21.6%. For time-resolved data Ⓑ, it was correctly predicted with 71.2% and falsely as *Burkholderia pyrrocinia* with 18.4%. For the combined data set Ⓒ, *Yersinia aldovae* was correctly predicted with 84.0%. The formerly prominent false predictions have dropped significantly to 11.2% as *Burkholderia pyrrocini* and 3.2% as *Burkholderia pyrrocinia*. *Oligella urethralis* was falsely predicted as *Burkholderia fungorum* with spectral data Ⓐ (10.4%). This is not the case for time-resolved data Ⓑ, where the erroneous assignments distribute over five different species. As a result, the overall false prediction rate based on the complete data set Ⓒ is reduced to 10.4% from 17.6% for Ⓐ and Ⓑ. *Burkholderia pyrrocinia*, with spectral data Ⓐ was falsely predicted as *Burkholderia fungorum* (12.0%) and *Yersinia aldovae* (12.8%). With time-resolved data this sample was incorrectly assigned as *Bacillus atrophaeus* (24.0%) and *Burkholderia fungorum* (13.6%). For the combined data set Ⓒ the incorrect predictions were reduced to 10.4% with no major falsely predicted species. In 17% of the cases *Burkholderia fungorum* was predicted as *Burkholderia pyrrocinia* with spectral data Ⓐ and as *Yersinia aldovae* with time-resolved data. With a combined data set Ⓒ, the incorrect prediction was significantly reduced to 13.6% as *Burkholderia pyrrocinia* and 8.8% as *Yersinia aldovae*. For all the discussed species, the correct prediction increased for the combined data set Ⓒ. In all cases, significant false predictions from Ⓐ and Ⓑ did not support each other, clearly indicating complementary information from Ⓐ and Ⓑ.

For the remaining substances the following observations can be made in Table 1:

- Compared to the spectral data, the prediction of *Micrococcus luteus* based on nothing but time-resolved data Ⓑ performs much better (>99%).
- The samples are grouped with bacteria in the first seven rows and columns, and oils in the last six columns and rows in each part of Table 1. For part Ⓐ and Ⓒ, no assignment between those groups is found (all other cells are equal to zero), i.e., a classification between oils and bacteria is possible with 100% accuracy. Even for the confusion matrix of time-resolved data Ⓒ, an excellent classification between oils and bacteria is possible with 99.6% accuracy.
- Fluorescence decay in Ⓑ yields enough information for a good discrimination. Time-dependent fluorescence data detected at 460 nm contain valuable information for a classification with nearly 82% accuracy. Highest misassignment can be found for the spectral curves which reveal only slight differences for some samples (especially for *Burkholderia fungorum*, *Burkholderia pyrrocinia* and *Yersinia aldovae*), as already seen from Figure 2.
- Comparing the diagonals in confusion matrices Ⓐ and Ⓒ, it should be noticed that each value for the full dataset in Ⓒ is higher than for the spectral data only in Ⓐ. Thus, additional information from the time-resolved data set not only improves the overall accuracy but supports a better classification of each measured sample.

## 4. Summary and Conclusions

The presented system is based on the detection of the fluorescence spectra (excited at two wavelengths) and of time-dependent fluorescence at a single channel (here 460 nm) for each excitation wavelength (here only 355 nm). It has been shown that information from time-resolved measurements and spectral data at least partly complement each other.

For the investigated group of samples, it has been demonstrated that fluorescence lifetime measured on a single emission channel (at 460 nm) provides sufficient information for sample classification higher than 80%. Partly, this information is orthogonal to spectral data. Thus, the overall accuracy of 86.5% of the classification algorithm was increased by more than 6% to 92.7% by combining spectral and temporal behavior of the fluorescence. In common work, classification accuracies are frequently given for groups of samples that are relevant for a specific scenario and application. Here, the classes within the sample set were defined on maximum depth, i.e., the 13 classes correspond to 13 agents. The confusion matrices in Table 1 contain the necessary information to calculate accuracies within the sample set. If e.g., the main goal for the measurement was to distinguish bacteria and oils, the accuracies of the classification algorithm were 100%, 99.6% and 100% for spectral, time-resolved and both data types, respectively.

Compared to pure chemicals, bacterial samples are complex due to their morphology, chemical structure and metabolism. Especially the LIF signals from For *Oligella urethralis* and *Yersinia aldovae* are predicted as *Burkolderia fungorum* and *Burkolderia pyrrocinia* for time-resolved and spectral measurements, respectively. From Table 1, the gain of a combined spectral and time-resolved data acquisition can be extracted. The number of mispredictions is reduced from 45 (spectral) and 36 (time-resolved) to 18, e.g., for *Yersinia aldovae*. This corresponds to an improved prediction rate from 64.0% to 85.6% for *Yersinia aldovae*. Within the group of oils, the samples Diesel 1 and Diesel 2, can be distinguished by the applied LIF technology with errors of 11%. The fluorescence process and thus the decay is sensitive to intra- and intermolecular energy transfer processes (e.g., quenching). It is expected that the complexity of fluorescence might become noticeable when mixed, impure, or contaminated samples are examined.

The design of the detection system offers reserves for further improvements such as additional time-dependent emission channels. This time-dependent approach is especially suited for LIF detection of bio-contaminated surfaces rather than spatially extended objects like aerosols when the travel time of a laser pulse through the cloud is larger than the fluorescence lifetime itself.

Future work will incorporate larger data sets (including both time-resolved and spectral data) of bacterial, chemical and background samples, followed by an analysis and evaluation of acquired spectral and temporal features of the data. From these results, an optimized design for a compact, robust and cost-effective detection system will be possible. Up to now, the sensitivity of the presented detection system promises to be a successful approach for measurements at increased standoff distances for security-related applications (staying in the eye-safe regime for the laser pulses).

## Figures and Tables

**Table 1:** Confusion matrices for classification of spectral, time-resolved and combined data in the upper, middle and lower part of the table, respectively. The left column indicates the accuracy of the model applied to the respective data set (see text).

Labeled as ↓		⟶ Predicted as												
		*B. atroph.*	*B. thur.*	*B. fung.*	*B. pyrr.*	*M. luteus*	*O. ureth.*	*Y. aldo.*	Anderol	Diesel 1	Diesel 2	kerosene	motor oil 1	motor oil 2
Ⓐ spectral (A=86.5%)	*B.atroph.*	121	1	0	0	3	0	0	0	0	0	0	0	0
	*B. thur.*	0	116	0	0	6	0	3	0	0	0	0	0	0
	*B. fung.*	0	2	86	21	1	7	8	0	0	0	0	0	0
	*B. pyrr.*	0	2	15	81	4	7	16	0	0	0	0	0	0
	*M. luteus*	3	5	4	6	102	0	5	0	0	0	0	0	0
	*O. ureth.*	0	0	13	5	0	103	4	0	0	0	0	0	0
	*Y. aldo.*	0	3	5	27	7	3	80	0	0	0	0	0	0
	Anderol	0	0	0	0	0	0	0	125	0	0	0	0	0
	Diesel 1	0	0	0	0	0	0	0	0	108	17	0	0	0
	Diesel 2	0	0	0	0	0	0	0	0	17	108	0	0	0
	kerosene	0	0	0	0	0	0	0	0	0	0	125	0	0
	motor oil 1	0	0	0	0	0	0	0	0	0	0	0	125	0
	motor oil 2	0	0	0	0	0	0	0	0	0	0	0	0	125
Ⓑ time-resolved (A=81.7%)	*B.atroph.*	91	0	14	17	0	3	0	0	0	0	0	0	0
	*B.thur.*	0	117	0	0	0	0	1	0	0	0	0	3	4
	*B.fung.*	10	0	78	12	0	3	22	0	0	0	0	0	0
	*B.pyrr.*	30	0	17	66	0	2	10	0	0	0	0	0	0
	*M.luteus*	0	0	0	0	124	1	0	0	0	0	0	0	0
	*O.ureth.*	8	0	3	6	4	103	1	0	0	0	0	0	0
	*Y.aldo.*	4	1	23	5	0	3	89	0	0	0	0	0	0
	Anderol	0	0	0	0	0	0	0	125	0	0	0	0	0
	Diesel 1	0	0	0	0	0	0	0	0	101	24	0	0	0
	Diesel 2	0	0	0	0	0	0	0	0	47	78	0	0	0
	kerosene	0	0	0	0	0	0	0	0	0	0	125	0	0
	motor oil 1	0	0	0	0	0	0	0	0	0	0	0	125	0
	motor oil 2	0	10	1	0	0	0	8	0	0	0	0	1	105
Ⓒ combined (A=92.6%)	*B.atroph.*	124	0	0	1	0	0	0	0	0	0	0	0	0
	*B.thur.*	0	125	0	0	0	0	0	0	0	0	0	0	0
	*B.fung.*	0	0	97	17	0	0	11	0	0	0	0	0	0
	*B.pyrr.*	0	0	15	96	0	3	11	0	0	0	0	0	0
	*M.luteus*	0	0	0	0	125	0	0	0	0	0	0	0	0
	*O.ureth.*	0	0	4	8	1	112	0	0	0	0	0	0	0
	*Y.aldo.*	0	1	14	4	0	1	105	0	0	0	0	0	0
	Anderol	0	0	0	0	0	0	0	125	0	0	0	0	0
	Diesel 1	0	0	0	0	0	0	0	0	111	14	0	0	0
	Diesel 2	0	0	0	0	0	0	0	0	15	110	0	0	0
	kerosene	0	0	0	0	0	0	0	0	0	0	125	0	0
	motor oil 1	0	0	0	0	0	0	0	0	0	0	0	125	0
	motor oil 2	0	0	0	0	0	0	0	0	0	0	0	0	125
